# Supplementary material for: SSRP1-mediated histone H1 eviction promotes replication origin assembly and accelerated development
Source: Nat Commun. 2020 Mar 12;11:1345. doi: 10.1038/s41467-020-15180-5 (PMC7067836; doi:10.1038/s41467-020-15180-5)
Supplement: Supplementary file 1 — Supplementary Information [file 41467_2020_15180_MOESM1_ESM.pdf]

Supplementary information for the manuscript

SSRP1-mediated histone H1 eviction promotes replication origin assembly and accelerated development

Lucia Falbo[1], Erica Raspelli[1], Francesco Romeo[1], Simona Fiorani[2], Federica Pezzimenti[1], Francesca Casagrande[1], Ilaria Costa[1], Dario Parazzoli[1] and Vincenzo Costanzo[1,3*]

[1]IFOM, The FIRC Institute of Molecular Oncology, Via Adamello 16, Milan 20139, Italy. [2]Clare Hall Laboratories, London Research Institute, Cancer Research UK, South Mimms, Herts EN63LD. [3]Department of Oncology and Haematology-Oncology, University of Milan, Milan, Italy.

*Corresponding author email: Vincenzo.Costanzo@ifom.eu

Supplementary Figure 1

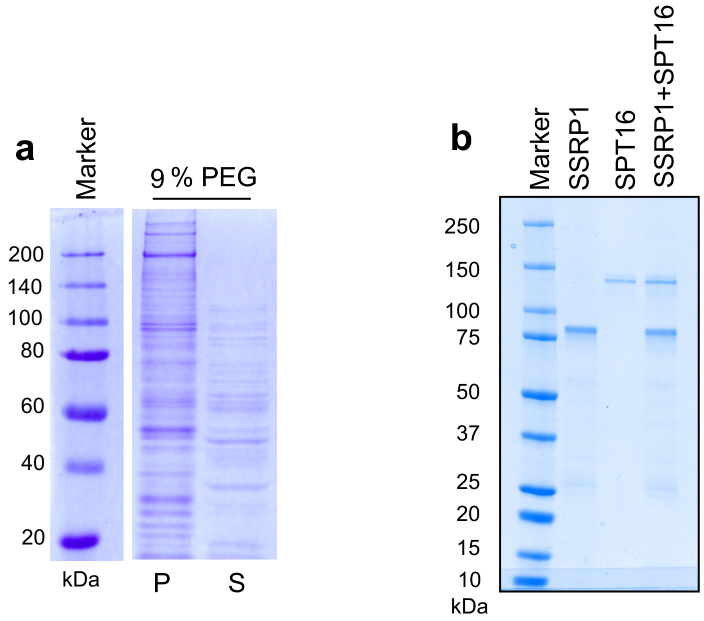

Supplementary Figure 1. Extract fractionation and recombinant SSRP1 and SPT16 proteins

a) Coomassie-stained SDS-PAGE gel of 9%P and 9%S fractions. **b**) Coomassie-stained SDS-PAGE gel of recombinant Flag-SSRP1, 6xHis-SPT16 and FACT complex (Flag-SSRP1+6xHis-SPT16) used throughout the manuscript.

Supplementary Figure 2

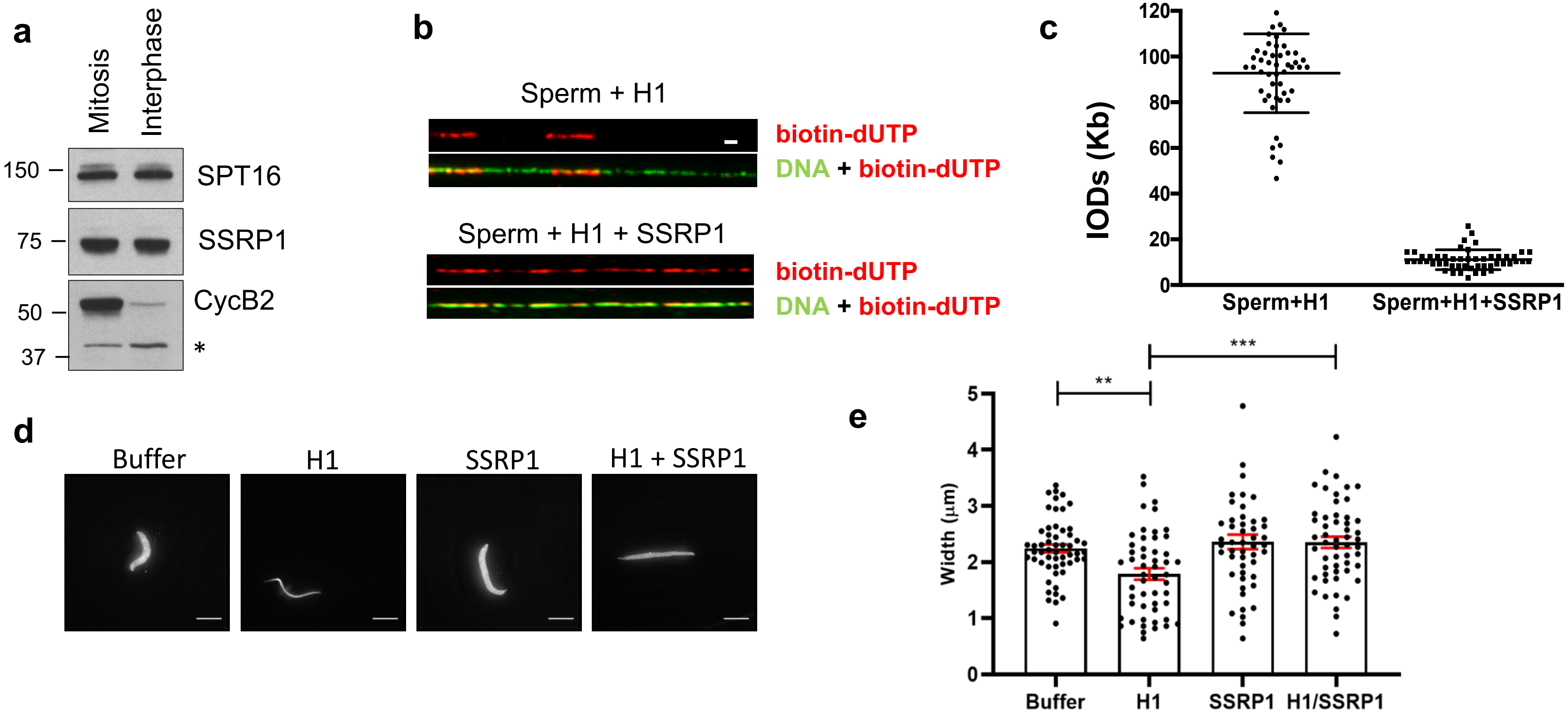

Supplementary Figure 2. SSRP1 counteracts histone H1 mediated inhibition of replication origin assembly

a) WB of the indicated proteins in mitotic and interphase extract. *indicates non-specific band. **b**) DNA combing assay showing biotin-dUTP incorporation in sperm nuclei pre-incubated with 2 μM recombinant histone H1 or 2 μM recombinant histone H1 plus 200 ng/ml SSRP1 and then transferred interphase egg extract. DNA was isolated and subjected to the combing procedure to visualize DNA fibers (green) and biotin-dUTP labeled tracts (red). Bar=10 Kb **c**) Graph shows individual IODs ± SEM for each sample, indicated in kb and calculated as in Fig 2. n=50 IODs; p<0.0001; unpaired *t* test. **d**) Examples of DAPI stained sperm nuclei pre-incubated with 2 μM recombinant histone H1, 200 ng/ml SSRP1 and histone H1 plus 200 ng/ml recombinant SSRP1 and then transferred to egg extract for 15 min. Bar= 10 mm. **e**) Graph showing width of single nuclei derived from the extracts treated as in (e). Bars represent mean ± SEM. n=50 nuclei; p<0.001; Two-way ANOVA.

Supplementary Figure 3

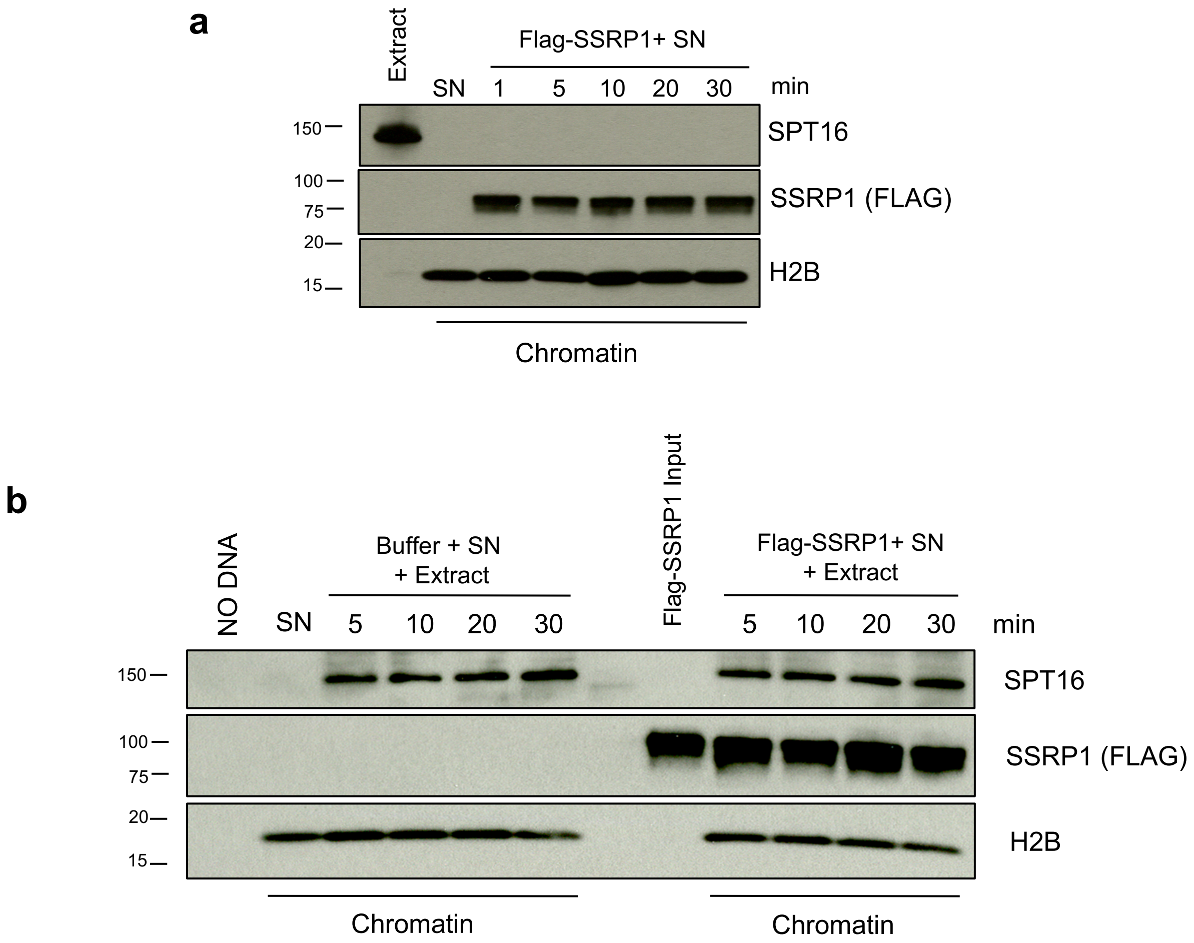

Supplementary Figure 3. SPT16 independent action of SSRP1 on somatic chromatin

a) WB of chromatin isolated from SN incubated for the indicated times with 200 ng/ml recombinant Flag-SSRP1. b) WB of chromatin isolated from SN pre-incubated with buffer or 200 ng/ml recombinant Flag-SSRP1 for 30 min, transferred to interphase egg extracts and incubated for the indicated times.

Supplementary Figure 4

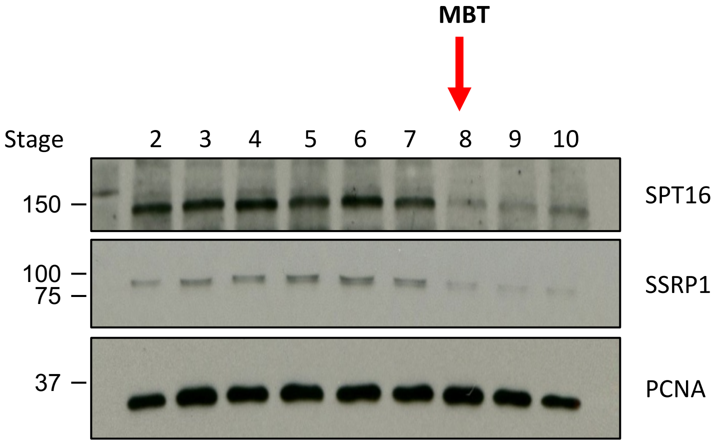

Supplementary Figure 4. SSRP1 and SPT16 decay at MBT

WB of whole embryos taken at the indicated stages using anti SSRP1, anti PCNA and anti SPT16 antibodies

Supplementary Figure 5

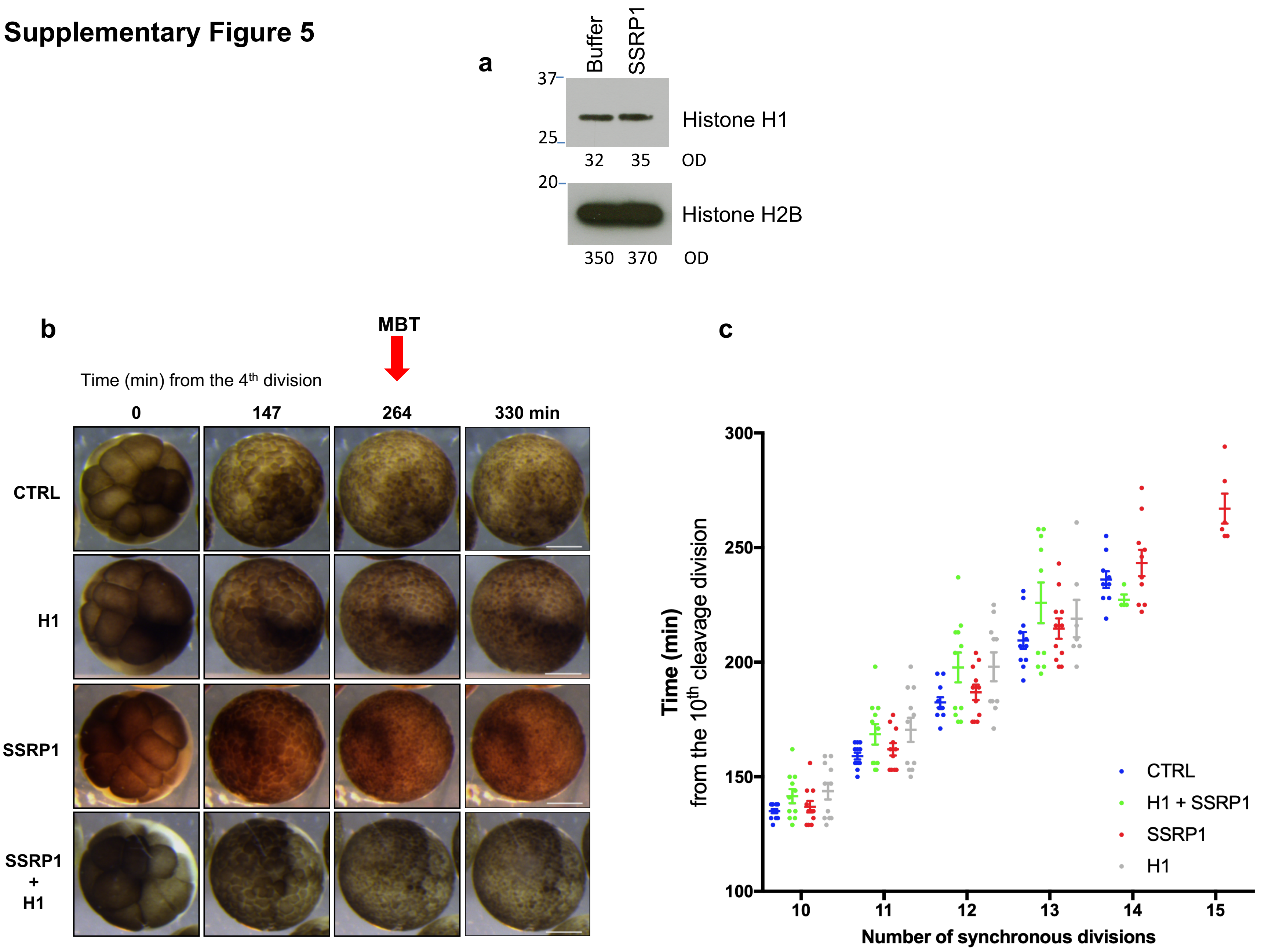

Supplementary Figure 5. Total levels of histone H1 in SSRP1 injected embryos and histone H1 mediated suppression of SSRP1 induced MBT delay

a)WB of buffer or SSRP1 injected post MBT embryos at stage 10 using anti histone H1 and anti histone H2A antibodies. Optical density (OD) is indicated. **b**) Time lapse video frames taken from a movie of developing embryos at the indicated times from the 4th cleavage set as time 0. Embryos were injected at the one-cell stage with control buffer (CTRL), histone H1, Myc-SSRP1 or histone H1 plus Myc-SSRP1 mRNA. Size bar=500 μm **c**) Graph showing the number of synchronous divisions-only after the 4th cleavage of 12 embryos injected as in (a) and followed up to 450 min from fertilization. Cleavages 1-9 were omitted. Bars show mean ± SEM; n=12 embryos

Supplementary Figure 6

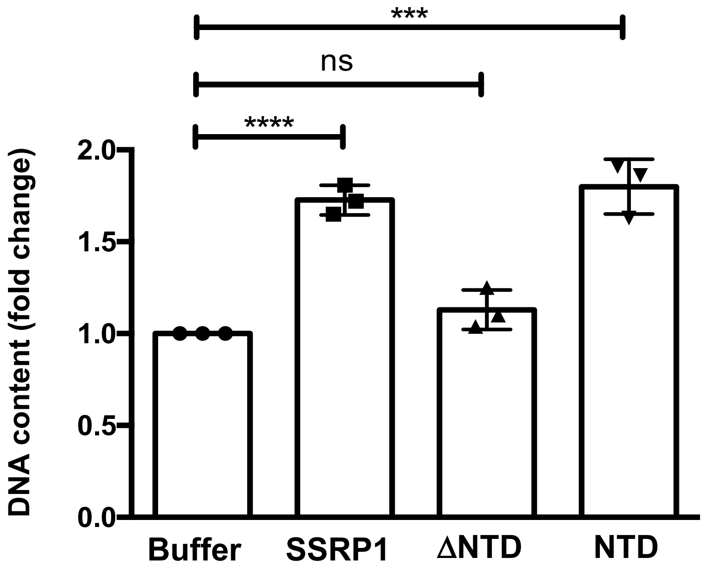

Supplementary Figure 6. SSRP1 mediated embryo DNA content increase

Fold change in DNA content in embryos injected with mRNAs indicated under each bar and collected at stage 10. DNA content in embryos injected with buffer was considered as 1. Bars represent mean ± SEM. n=3 experiments; ****p<0.0001; ***p<0.001; unpaired *t* test.

Supplementary Figure 7

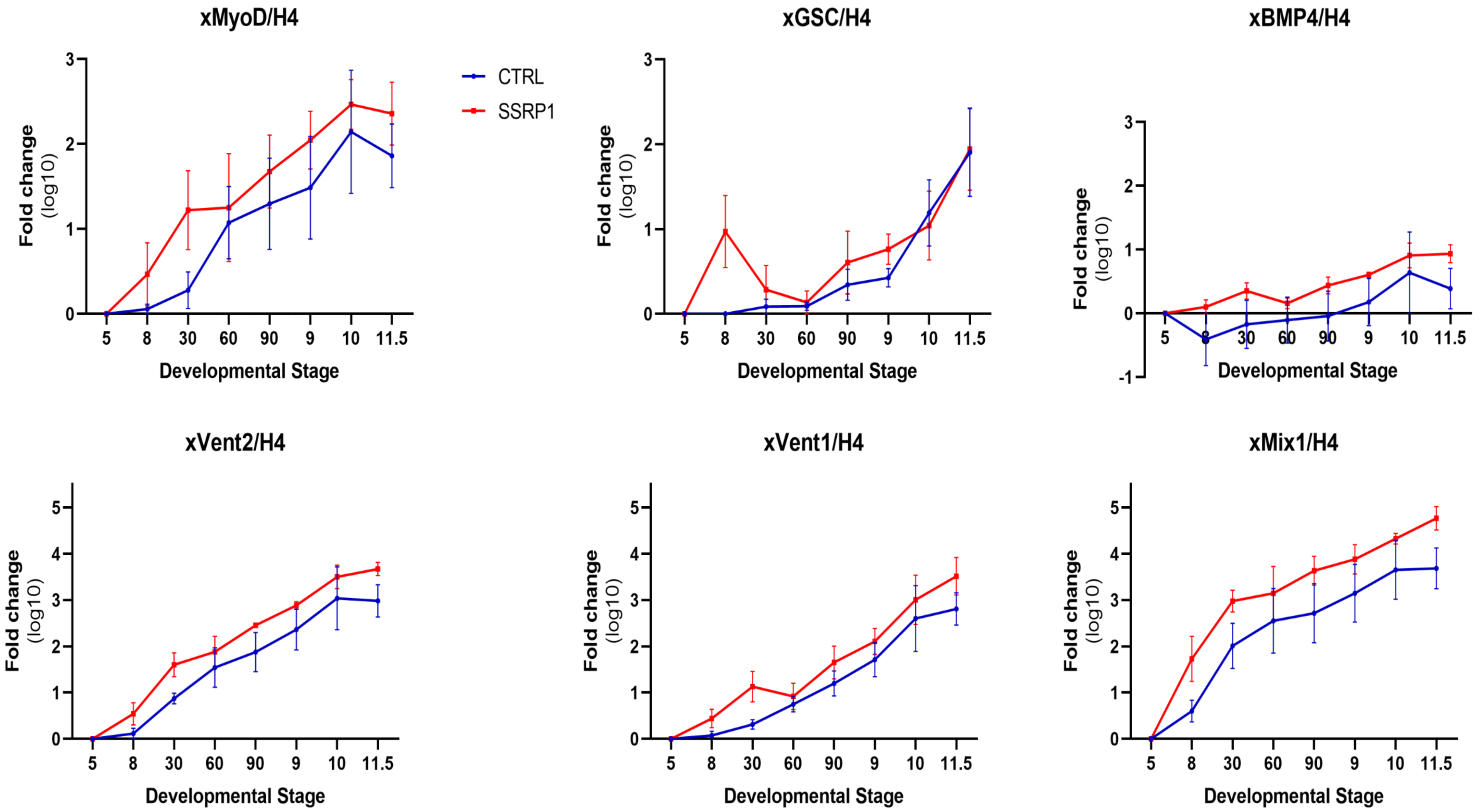

Supplementary Figure 7. SSRP1 action on early transcribed genes in post MBT embryos

Real time PCR quantification of mRNA levels of the indicated genes expressed at different developmental stages in embryos injected with buffer (CTRL) or Myc-SSRP1 mRNA and normalized to histone H4 levels. n=3 independent experiments; mean values ± SEM are shown.

Supplementary Figure 8

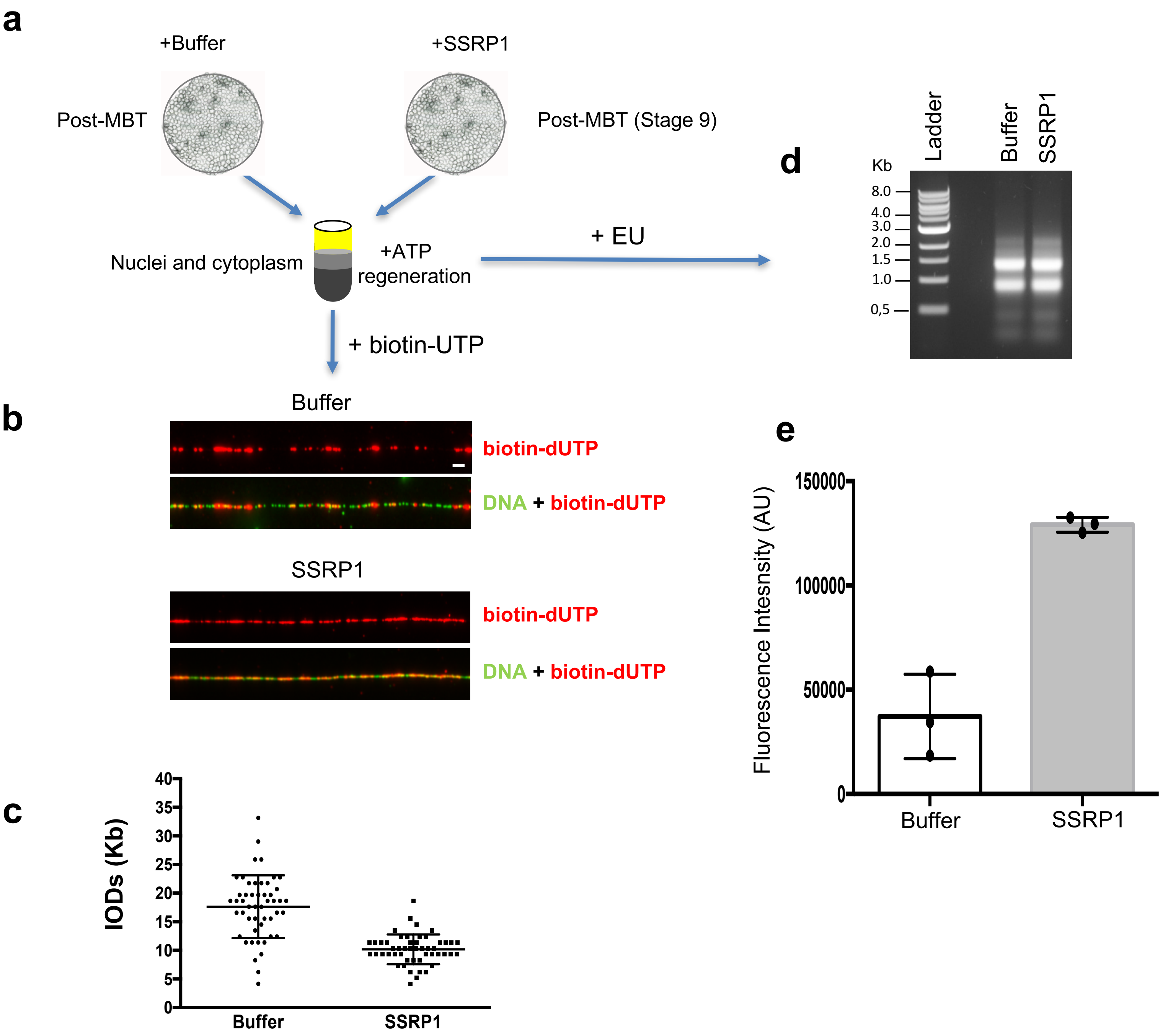

Supplementary Figure 8. Nucleo-cytoplasmic extracts derived from developing embryos showing *in vivo* effects of SSRP1 on DNA replication origin assembly and transcription

a) Scheme showing production of nucleo-cytoplasmic extracts from embryos at stage 9 injected with buffer or Myc-SSRP1. See methods for details. **b**) DNA combing assay showing biotin-dUTP incorporation in nuclei isolated from embryos at stage 9 injected with Buffer or Myc-SSRP1. DNA was isolated and subjected to combing procedure to visualize DNA template fibers (green) and biotin-dUTP labeled tracts corresponding to origins of DNA replication (red). Bar=10 Kb. **c**) Graph showing IODs indicated in kb and calculated as in Fig 2. Bar indicates mean ± SEM; n=50 IODs; $p<0.0001$; unpaired *t* test. **d**) Ethidium bromide stained agarose gel showing total RNA extracted from nucleo-cytoplasmic extracts prepared as in (a) from 100 post-MBT embryos at stage 9 injected with buffer or Myc-SSRP1 mRNA. **e**) Fluorescence intensity of *de novo* transcribed RNA obtained by incubating nucleo-cytoplasmic extracts with Ethynyl-Uridine (EU). RNA was extracted as in (d) from buffer or Myc-SSRP1 mRNA embryos. EU containing RNA was labeled with Alexa Fluor 594. Bar indicates mean ± SEM. n=3 independent experiments; $p<0.01$; unpaired *t*-test

Supplementary Figure 9

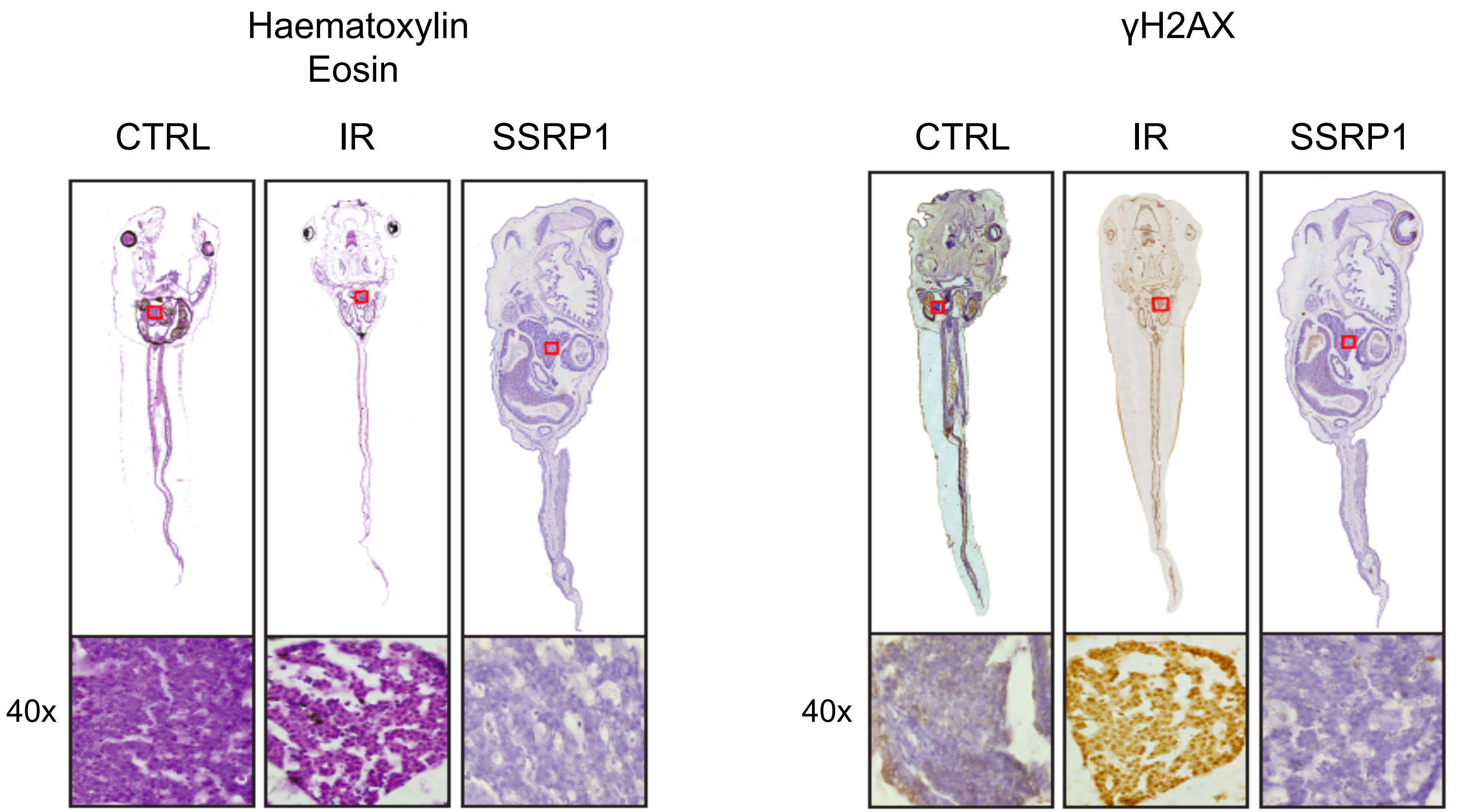

Supplementary Figure 9. Absence of DNA double strand breaks in SSRP1 treated embryos

Histology of *Xenopus* tadpoles collected at stage 47. Frontal-sections from head to tail of embryos that were injected with buffer (CTRL), SSRP1 mRNA (SSRP1) or were irradiated with ionizing radiation (IR) (5 Gys). Embryos were stained with hematoxylin and eosin (left panel) and anti phospho-H2AX (right panel). Details at higher magnification (40x) are shown in the inset.
