# Supplementary material for: SSRP1-mediated histone H1 eviction promotes replication origin assembly and accelerated development
Source: Nat Commun. 2020 Mar 12;11:1345. doi: 10.1038/s41467-020-15180-5 (PMC7067836; doi:10.1038/s41467-020-15180-5)
Supplement: Supplementary file 3 — Description of Additional Supplementary Files [file 41467_2020_15180_MOESM3_ESM.pdf]

Description of Additional Supplementary Files

File Name: Supplementary Data 1
Description: Proteins enriched in the fraction stimulating somatic nuclei replication

File Name: Supplementary Movie 1
Description: Time-lapse movie of twelve individual developing embryos. The top panel shows embryos injected with buffer. The lower panel shows embryos injected with Myc-SSRP1 mRNA.

File Name: Supplementary Movie 2
Description: Time-lapse movie of twelve individual developing embryos. The top panel shows embryos injected with buffer. The lower panel shows embryos injected with Myc-ΔNTD mRNA.

File Name: Supplementary Movie 3
Description: Time-lapse movie of twelve individual developing embryos. The top panel shows embryos injected with buffer. The lower panel shows embryos injected with Myc-NTD mRNA.
