# Supplementary material for: SSRP1-mediated histone H1 eviction promotes replication origin assembly and accelerated development
Source: Nat Commun. 2020 Mar 12;11:1345. doi: 10.1038/s41467-020-15180-5 (PMC7067836; doi:10.1038/s41467-020-15180-5)
Supplement: Supplementary file 8 — Reporting Summary [file 41467_2020_15180_MOESM8_ESM.pdf]

nature research

Corresponding author(s): Vincenzo Costanzo

Last updated by author(s): 27/01/2020

Reporting Summary

Nature Research wishes to improve the reproducibility of the work that we publish. This form provides structure for consistency and transparency in reporting. For further information on Nature Research policies, see Authors & Referees and the Editorial Policy Checklist.

Statistics

For all statistical analyses, confirm that the following items are present in the figure legend, table legend, main text, or Methods section.

n/a	Confirmed	
☐	☒	The exact sample size (*n*) for each experimental group/condition, given as a discrete number and unit of measurement
☐	☒	A statement on whether measurements were taken from distinct samples or whether the same sample was measured repeatedly
☐	☒	The statistical test(s) used AND whether they are one- or two-sided<br>*Only common tests should be described solely by name; describe more complex techniques in the Methods section.*
☐	☒	A description of all covariates tested
☒	☐	A description of any assumptions or corrections, such as tests of normality and adjustment for multiple comparisons
☐	☒	A full description of the statistical parameters including central tendency (e.g. means) or other basic estimates (e.g. regression coefficient) AND variation (e.g. standard deviation) or associated estimates of uncertainty (e.g. confidence intervals)
☒	☐	For null hypothesis testing, the test statistic (e.g. *F*, *t*, *r*) with confidence intervals, effect sizes, degrees of freedom and *P* value noted<br>*Give P values as exact values whenever suitable.*
☒	☐	For Bayesian analysis, information on the choice of priors and Markov chain Monte Carlo settings
☒	☐	For hierarchical and complex designs, identification of the appropriate level for tests and full reporting of outcomes
☒	☐	Estimates of effect sizes (e.g. Cohen's *d*, Pearson's *r*), indicating how they were calculated

Our web collection on statistics for biologists contains articles on many of the points above.

Software and code

Policy information about availability of computer code

Data collection	N/A
Data analysis	N/A

For manuscripts utilizing custom algorithms or software that are central to the research but not yet described in published literature, software must be made available to editors/reviewers. We strongly encourage code deposition in a community repository (e.g. GitHub). See the Nature Research guidelines for submitting code & software for further information.

Data

Policy information about availability of data

All manuscripts must include a data availability statement. This statement should provide the following information, where applicable:
- Accession codes, unique identifiers, or web links for publicly available datasets
- A list of figures that have associated raw data
- A description of any restrictions on data availability

Data supporting the findings of this manuscript have all been included. A separate source data file contains raw data underlying Fig 1a-b, 1d-e, 2a-g, 3a-d, 4a, 4c, 5a-f, 6b-c, 6e-f, 6h-i, 7b-c, 7e-f, 7h-i and Supplementary Figs 2a, 2c, 2e, 3a-b, 4, 5a, 5c, 6, 7 and 8c-e.

Field-specific reporting

Please select the one below that is the best fit for your research. If you are not sure, read the appropriate sections before making your selection.

☒ Life sciences ☐ Behavioural & social sciences ☐ Ecological, evolutionary & environmental sciences

Life sciences study design

All studies must disclose on these points even when the disclosure is negative.

Sample size	We did not use any statistical method to determine sample size. Sample size was chosen accordingly to previous publications.
Data exclusions	N/A
Replication	Experiments were repeated at least three times to confirm reproducibility.
Randomization	N/A
Blinding	N/A

Reporting for specific materials, systems and methods

We require information from authors about some types of materials, experimental systems and methods used in many studies. Here, indicate whether each material, system or method listed is relevant to your study. If you are not sure if a list item applies to your research, read the appropriate section before selecting a response.

Materials & experimental systems

n/a	Involved in the study	
☐	☒	Antibodies
☒	☐	Eukaryotic cell lines
☒	☐	Palaeontology
☐	☒	Animals and other organisms
☒	☐	Human research participants
☒	☐	Clinical data

Methods

n/a	Involved in the study	
☒	☐	ChIP-seq
☒	☐	Flow cytometry
☒	☐	MRI-based neuroimaging

Antibodies

Antibodies used	were probed using the following antibodies obtained from J. Gannon (The Francis Crick Institute, London, UK)(dilutions are indicated in parenthesis): rabbit polyclonal anti-Xenopus Cdc45 (1:2000), mouse monoclonal anti-Xenopus Orc1 (1:100000); rabbit polyclonal anti-Xenopus Orc2 (1:4000), mouse monoclonal anti-Cdk1 (A17, 1:500), mouse monoclonal anti-Cdk1 pTyr15 (1:1000), mouse monoclonal anti-Xenopus Cyclin B2 (X121, 1:500). Rabbit polyclonal anti-Xenopus H2A.X-F was obtained from D. Shechter Lab (Albert Einstein college of medicine) and used at 1:10,000 dilution. We also used the following commercial antibodies (source, catalogue and dilutions are indicated in parenthesis): anti-MCM7 (47DC141, Santa Cruz Biotechnology, sc-9966, 1:4000), anti-PCNA (PC10, Serotec, MCA1558, 1:5000), mouse monoclonal anti-Cdt1 (F-6, Santa Cruz Biotechnology, sc-365305, 1:500), mouse monoclonal anti-Ssrp1 (10D7, Abcam, ab26212, 1:1000), rabbit polyclonal anti-Ssrp1 (A303-068A, Bethyl Laboratories, 1:1000), rabbit polyclonal anti-Spt16 (H-300, Santa Cruz Biotechnology, sc-28734, 1:250), rabbit polyclonal anti-Cdc6 (Santa Cruz Biotechnology, sc-8341, 1:1000), mouse monoclonal anti-Xenopus Polα (p180) Abmart (clone 13026-1-3/C199, 1:500), mouse monoclonal anti-H1.0 (Abcam, ab11079, 1:1000), rabbit polyclonal anti-H2B (Millipore, 07-371, 1:1000), mouse monoclonal anti-FLAG-M2-Peroxidase (HRP) (A8592, Sigma, 1:1000) and rabbit polyclonal anti-SMC2 (Bethyl, A300-056A, 1:1000). Detection with secondary antibodies was commonly carried out at 1:10,000 using ECL detection reagents (GE) or WesternBright ECL (Advansta) on Amersham Hyperfilm (GE) or Kodak.
Validation	Antibodies were described and validated in this and previous publications referenced in the manuscript

Animals and other organisms

Policy information about studies involving animals; ARRIVE guidelines recommended for reporting animal research

Laboratory animals	Adult Xenopus laevis female and male frogs provided by NASCO
Wild animals	N/A
Field-collected samples	N/A
Ethics oversight	Use of Xenopus laevis was approved by IFOM Animal Welfare committee and the Italian Ministry of Health. Part of the experiments were conducted also at Clare Hall Laboratories, London Research Institute under institute and Home Office authorization for the use of amphibians.

Note that full information on the approval of the study protocol must also be provided in the manuscript.
